# Pharynx mitochondrial [Ca^2+^] dynamics in live *C. elegans* worms during aging

**DOI:** 10.18632/oncotarget.18600

**Published:** 2017-06-22

**Authors:** Pilar Alvarez-Illera, Paloma García-Casas, Jessica Arias-del-Val, Rosalba I. Fonteriz, Javier Alvarez, Mayte Montero

**Affiliations:** ^1^ Department of Biochemistry and Molecular Biology and Physiology, Institute of Biology and Molecular Genetics, Faculty of Medicine, University of Valladolid and CSIC, Valladolid, Spain

**Keywords:** calcium, pharynx, C. elegans, aging, mitochondria, Gerotarget

## Abstract

Progressive decline in mitochondrial function is generally considered one of the hallmarks of aging. We have expressed a Ca^2+^ sensor in the mitochondrial matrix of *C. elegans* pharynx cells and we have measured for the first time mitochondrial [Ca^2+^] ([Ca^2+^]_M_) dynamics in the pharynx of live *C. elegans* worms during aging. Our results show that worms stimulated with serotonin display a pharynx [Ca^2+^]_M_ oscillatory kinetics that includes both high frequency oscillations (up to about 1Hz) and very prolonged “square-wave” [Ca^2+^]_M_ increases, indicative of energy depletion of the pharynx cells. Mitochondrial [Ca^2+^] is therefore able to follow “beat-to-beat” the fast oscillations of cytosolic [Ca^2+^]. The fast [Ca^2+^]_M_ oscillations kept steady frequency values during the whole worm life, from 2 to 12 days old, but the height and width of the peaks was progressively reduced. [Ca^2+^]_M_ oscillations were also present with similar kinetics in respiratory chain complex I *nuo-6* mutant worms, although with smaller height and frequency than in the controls, and larger width. In summary, Ca^2+^ fluxes in and out of the mitochondria are relatively well preserved during the *C. elegans* life, but there is a clear progressive decrease in their magnitude during aging. Moreover, mitochondrial Ca^2+^ fluxes were smaller in *nuo-6* mutants with respect to the controls at every age and decreased similarly during aging.

## INTRODUCTION

Mitochondrial dysfunction constitutes one of the more distinctive characteristics of aging [1–6]. In human muscle, the activity of the mitochondrial enzymes responsible for energy production has been shown to decline progressively with age [2], accompanied by reduction in mitochondrial volume, altered mitochondrial morphology, increased oxidative stress, compromised proteostasis and accumulation of mitochondrial DNA mutations [7–10]. Similar phenomena have been observed in the nematode *C. elegans*. Body wall muscle mitochondria undergo progressive fragmentation with aging, together with a decrease in mitochondrial length and area, and an increase in mitochondrial circularity [4]. The mechanism and functional significance of this progressive mitochondrial alteration is still obscure, particularly because a series of *C. elegans* mutants with mild mitochondrial alterations have extended lifespan [11]. This apparent paradox - aging is accompanied by mitochondrial alterations, but mitochondrial alterations promote survival - is still unresolved, but probably depends on the activation of specific survival pathways following early mitochondrial alteration [12–16].

Mitochondria have a central role in cellular energetics and ATP production, and one of the main modulators of these pathways is the Ca^2+^ ion. When cells are activated, the [Ca^2+^] rises first in the cytosol and then enters the mitochondria through the mitochondrial Ca^2+^ uniporter, activating several key dehydrogenases to boost substrate oxidation and ATP production [17–19]. Then, when cells return to the resting state, Ca^2+^ is extruded from mitochondria through Na^+^/Ca^2+^ or H^+^/Ca^2+^ exchangers [20, 21]. This transient mitochondrial Ca^2+^ uptake is driven by the mitochondrial membrane potential, and therefore reflects in some way the functional state of mitochondria. In addition, the exchangers responsible of Ca^2+^ release from mitochondria are thought to be electrogenic (i.e., stoichiometry 3Na^+^/1Ca^2+^) and thus they are also driven by the membrane potential. Therefore, both mitochondrial Ca^2+^ uptake and release are energized by the mitochondrial membrane potential.

We have decided to monitor mitochondrial [Ca^2+^] ([Ca^2+^]_M_) dynamics in *C. elegans* pharynx *in vivo* during aging, in order to study if the progressive mitochondrial alteration induces changes in [Ca^2+^]_M_ dynamics. We have targeted a yellow cameleon YC3.60 Ca^2+^ sensor [22] to the mitochondrial matrix of *C. elegans* pharynx using the myo-2 promoter and a mitochondrial targeting presequence. Using this probe we have measured for the first time mitochondrial Ca^2+^ dynamics in *C. elegans* pharynx, both in wild type worms and in mitochondrial complex I *nuo-6* mutants. Our data show that [Ca^2+^]_M_ is able to undergo fast oscillations during pharynx pumping, with a frequency similar to the cytosolic [Ca^2+^] oscillations ([Ca^2+^]_C_) we have previously reported [23]. In addition, prolonged “square-wave” [Ca^2+^]_M_ elevations were also observed, similar to those we have previously described in cytosolic [Ca^2+^] [23], and they were also more frequent in young worms. [Ca^2+^]_M_ oscillations were observed with similar frequency along the whole life of the worms, both in control worms and in *nuo-6* mutants, although both width and height decreased with age. In addition, [Ca^2+^]_M_ oscillations in *nuo-6* mutant worms showed reduced height and frequency, and increased width, compared with wild-type worms.

## RESULTS

### Generation of the mitochondrially-targeted Ca^2+^ sensor

**Figure 1:**
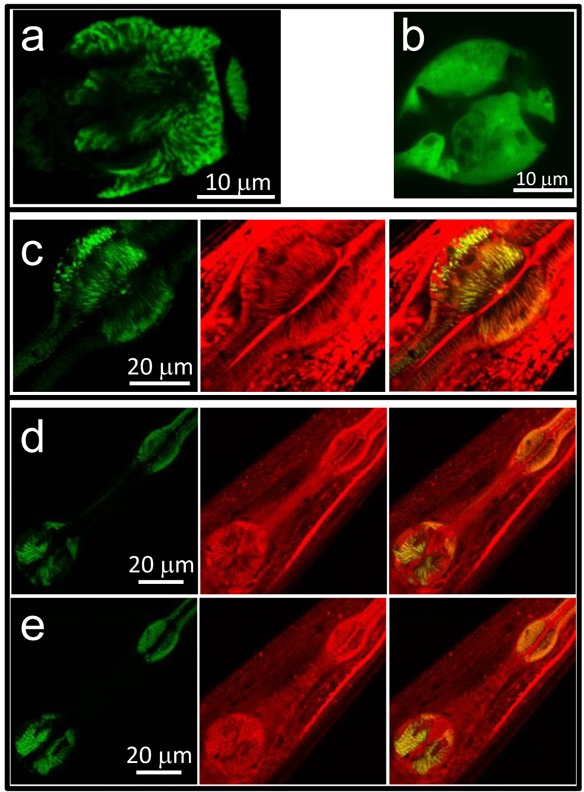
Confocal images of AQ3055, AQ3059 and *Mnuo-6* worms Panel **a.** shows an image of the fluorescence of the terminal bulb in an AQ3055 worm expressing the mitochondrially-targeted YC3.60 Ca^2+^ sensor. Panel **b.** shows an image of the fluorescence of the terminal bulb in an AQ3059 worm expressing the cytosolic YC3.60 Ca^2+^ sensor. Panel **c.** shows images of the AQ3055 worm loaded with mitotracker deep red. The images show the YC3.60 fluorescence (left) the mitotracker deep red fluorescence (middle) and the merge of both (right). Panels **d.** and **e.** show two confocal planes of a *Mnuo-6* worm loaded with mitotracker deep red. The images show in each case the YC3.60 fluorescence (left) the mitotracker deep red fluorescence (middle) and the merge of both (right).

To monitor mitochondrial [Ca^2+^], control N2 *C. elegans* nematodes were microinjected with the plasmid for expression of mitochondrially-targeted YC3.60 in pharynx under the myo-2 promoter. The transformed strain, named AQ3055, showed fluorescence in the pharynx with a typical striped mitochondrial distribution when analyzed by confocal microscopy (Figure 1a). Note the difference with the more homogeneous fluorescence of the cytosolic YC3.60 sensor expressed under the same promoter (Figure 1b). In addition, this fluorescence colocalized with that of mitotracker deep red (Figure 1c), indicating a correct mitochondrial localization of the Ca^2+^ sensor. Note that YC3.60 labels only pharynx mitochondria, while mitotracker deep red is not specific for the pharynx and labels mitochondria in every cell.

We have also studied mitochondrial Ca^2+^ signaling in respiratory chain complex I *nuo-6* mutants. *nuo-6* mutants expressing the mitochondrially-targeted Ca^2+^-sensitive protein (named here as *Mnuo-6*) were obtained by crossing AQ3055 worms with *nuo-6* (qm200) mutants [24], obtained from the Caenorhabditis Genetics Center. Figures 1d and 1e show two confocal planes of the YC3.60 fluorescence in the *Mnuo-6* mutants, showing also a typical mitochondrial pattern, which colocalized with mitotracker deep red. Therefore, the Ca^2+^ sensor is correctly targeted to the mitochondria also in this strain.

### Mitochondrial Ca^2+^ dynamics in wild-type worms during aging

**Figure 2:**
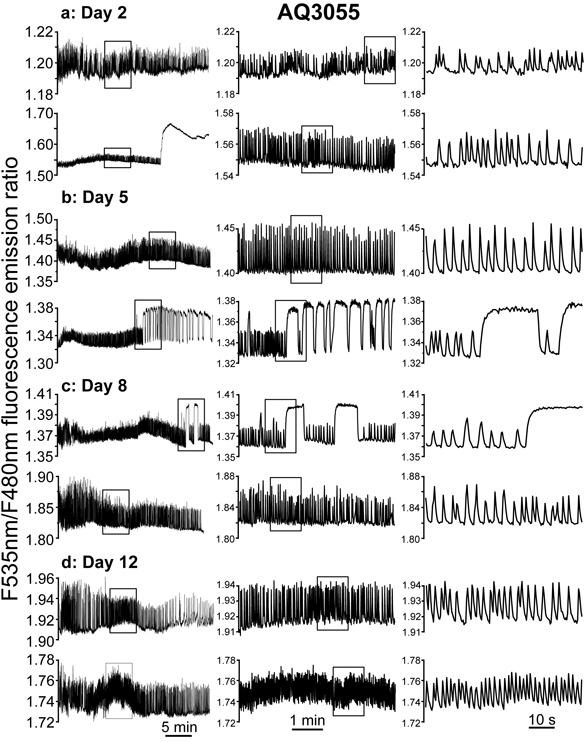
Representative traces of [Ca^2+^]_M_ dynamics in pharynx of *C. elegans* AQ3055 (N2 worms expressing the mitochondrial Ca^2+^ sensor) worms of 2 panel **a.**, 5 panel **b.**, 8 panel **c.** and 12 panel **d.** days old. Each panel shows two typical experiments performed in worms of a given age. The insets in the left panels are shown expanded in the middle panels, and the insets in the middle panels are shown expanded in the right panels.

[Ca^2+^]_M_ was first monitored in the AQ3055 *C. elegans* strain, in live worms glued to the agar pad and subjected to stimulation of pharynx pumping with serotonin, as described previously [23]. Figure 2 shows several representative traces of [Ca^2+^]_M_ dynamics monitored continuously at 2.5Hz for 30 min in live worms of 2, 5, 8 and 12 days old. The left panels show typical 30 min records of [Ca^2+^]_M_ dynamics obtained in different worms, two of them for every age. The middle panels show 5 min records taken each one from the insets in the corresponding left panels expanded. The right panels show 1 min records taken again each one from the insets in the corresponding middle panel expanded. The traces show that [Ca^2+^]_M_ undergoes rapid and persistent oscillations similar to those we have previously described in cytosolic [Ca^2+^] [23]. In addition, some worms had large and persistent increases in [Ca^2+^]_M_, that in some cases could last for many minutes until the end of the experiment (see 2^nd^ trace of day 2) or in other cases could be shorter and reversible (see 2^nd^ trace of day 5 and 1^st^ trace of day 8). Similar prolonged increases in [Ca^2+^]_M_ were also found in cytosolic [Ca^2+^], where we called them “square-wave” [Ca^2+^] increases [23]. Therefore, cytosolic and mitochondrial [Ca^2+^] dynamics behave similarly, suggesting that [Ca^2+^]_M_ in the pharynx directly follows the dynamics of cytosolic [Ca^2+^], responding with fast [Ca^2+^]_M_ oscillations when cytosolic [Ca^2+^] is oscillating, and with persistent increases in [Ca^2+^]_M_ when cytosolic [Ca^2+^] keeps persistently high.

To obtain more information on the complex [Ca^2+^]_M_ dynamics data obtained, we have made a series of experiments like those shown in Figure 2 in 29-35 worms of every age, and then we have made a detailed computer analysis of the main characteristics of each Ca^2+^ transient measured. As described previously [23], our analysis provides information on the width, the height and the frequency of every [Ca^2+^] peak in our experiments. Then, we can pool together all the data corresponding to every age and follow the evolution with the age of every parameter.

**Figure 3:**
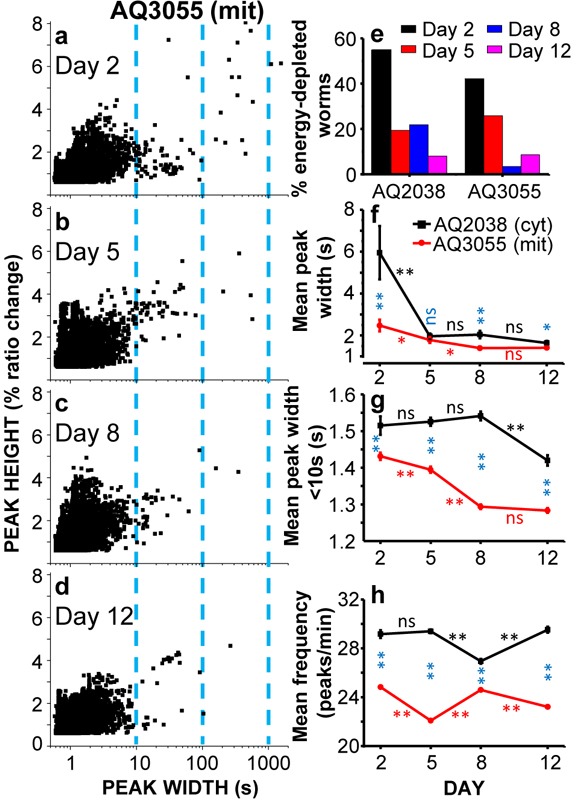
Comparative analysis of [Ca^2+^]_C_ and [Ca^2+^]_M_ dynamics in AQ2038 (N2 worms expressing the cytosolic [Ca^2+^] sensor) or AQ3055 (N2 worms expressing the mitochondrial Ca^2+^ sensor) worms of 2, 5, 8 and 12 days old, at 2.5Hz image resolution Panels a-d show plots of peak height against peak width for all the [Ca^2+^]_M_ peaks analyzed in AQ3055 worms of 2 (panel **a.**, 8414 peaks from 38 worms studied), 5 (panel **b.**, 9365 peaks from 29 worms studied), 8 (panel **c.**, 10802 peaks from 29 worms studied) and 12 (panel **d.**, 11232 peaks from 35 worms studied) days old. Panel **e.** shows the percentage of worms considered as energy-depleted because the [Ca^2+^]_c_ or [Ca^2+^]_M_ record shows or ends in prolonged “square wave” [Ca^2+^] peaks. Panels **f.**-**h.** show the variation with worm age of the main parameters analyzed in the Ca^2+^ imaging experiments performed in AQ2038 and AQ3055 worms: mean peak width (panel f), mean peak width considering only peaks with width < 10s (panel g) and mean peak frequency (panel h). Significance was obtained by the ANOVA test (*, *p* < 0,05; **, *p* < 0,001; ns, not significant) comparing: i) the [Ca^2+^]_C_ and [Ca2+]M data of the same day (blue symbols), ii) the [Ca^2+^]_C_ data of every two consecutive days (black symbols) and iii) the [Ca^2+^]_M_ data of every two consecutive days (red symbols).

Figure 3, panels a-d, shows plots of peak height against peak width for all the [Ca^2+^]_M_ peaks obtained in AQ3055 worms of day 2, 5, 8 and 12 of adult life. In this plot, the prolonged “square-wave” [Ca^2+^]_M_ peaks correspond to the points having larger width, those placed at the right of every graph (note the logarithmic scale). As previously shown for the cytosolic [Ca^2+^] peaks, the proportion of long “square-wave” [Ca^2+^]_M_ peaks was higher in young worms (day 2) and decreased progressively as they get older (day 12). As shown before for cytosolic [Ca^2+^] [23], many of these long [Ca^2+^]_M_ peaks started at some point along the experiment and stayed at high [Ca^2+^] until the end of the experiment (as shown in Figure 2, 2^nd^ trace of day 2). We have hypothesized that these long peaks are due to energy depletion of the pharynx cells, although paradoxically they occur more frequently in younger worms. Figure 3e shows the variation of the percentage of worms having long “square-wave” [Ca^2+^]_c_ (AQ2038 strain) or [Ca^2+^]_M_ (AQ3055 strain) peaks during aging. Very similar values were obtained when either [Ca^2+^]_c_ or [Ca^2+^]_M_ was measured. This could be expected, because a persistent increase in [Ca^2+^]_c_ should also induce a large [Ca^2+^] accumulation inside mitochondria. The [Ca^2+^]_c_ data in this and the following panels correspond to experiments performed in the AQ2038 strain at 2.5Hz image rate.

The decrease with the age in the number of long [Ca^2+^]_M_ peaks can be seen also when we calculate the mean width of all the [Ca^2+^]_M_ peaks obtained at every age. Panel f shows the evolution of the mean peak width with worm age, comparing the data obtained in pharynx cytosolic [Ca^2+^] in the AQ2038 strain with those obtained in pharynx mitochondrial [Ca^2+^] in the AQ3055 strain. The mean peak width of [Ca^2+^]_M_ peaks was smaller than the corresponding one of [Ca^2+^]_C_ at every age, but it followed the same decreasing trend with age. This decrease was mainly due to the disappearance of the long “square-wave” [Ca^2+^] peaks, because when the mean width was calculated using only the [Ca^2+^] peaks having a width smaller than 10s (Figure 3g), the changes with age were much smaller. In any case, the mean peak width of the [Ca^2+^]_M_ peaks was always smaller than that of the [Ca^2+^]_C_ peaks and decreased further during aging. Part of this effect may be due to the fact that mitochondria takes up Ca^2+^ from the cytosol with low Ca^2+^-affinity, hence mitochondrial Ca^2+^ uptake only occurs when [Ca^2+^]_c_ is above certain values.

The frequency of the [Ca^2+^] peaks was little modified with age, both in the case of [Ca^2+^]_C_ and [Ca^2+^]_M_ measurements (Figure 3h). However, the frequency of the [Ca^2+^]_M_ peaks was about 20% smaller than that of the [Ca^2+^]_C_ peaks at every age. This may probably be due to the presence of [Ca^2+^]_C_ peaks which are too small to evoke a significant [Ca^2+^]_M_ response.

### Mitochondrial Ca^2+^ dynamics in mutant *nuo-6* worms

**Figure 4:**
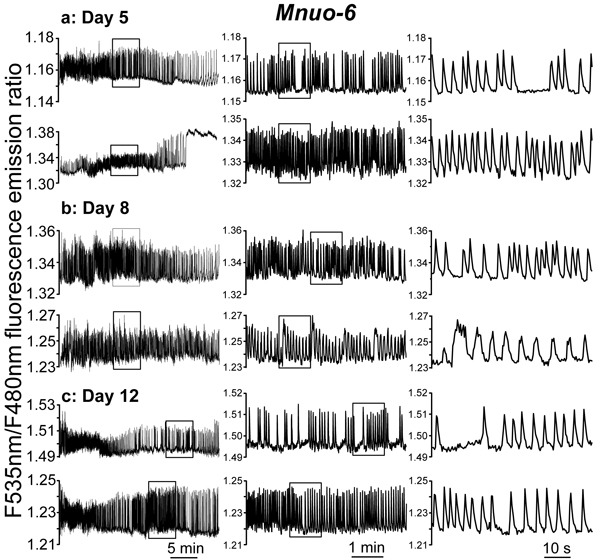
Representative traces of [Ca^2+^]_M_ dynamics in pharynx of *C. elegans Mnuo-6* worms (*nuo-6* mutant worms expressing the mitochondrial Ca^2+^ sensor) of 5 panel **a.**, 8 panel **b.** and 12 panel **c.** days old. Each panel shows two typical traces obtained in worms of a given age, using different time scales in the middle and right panels to expand the insets and appreciate better the dynamics.

We have then performed similar experiments with *nuo-6* respiratory chain mutants (*Mnuo-6* strain) expressing the mitochondrial [Ca^2+^] probe. Probably because of the slower rate of development of these mutant worms, the level of fluorescence of the probe was very small at day 2 of adult life, so that measurements were too noisy at that age. By this reason, in this strain we have performed measurements only at days 5, 8 and 12 of adult life. Figure 4 shows a series of typical [Ca^2+^]_M_ records obtained in this mutant strain at 5, 8 and 12 days of adult life. The traces show that mitochondrial Ca^2+^ oscillations are still present and are apparently very similar to the control ones (compare Figures 2 and 4). In addition, some prolonged “square wave” [Ca^2+^]_M_ increases could also be observed (see 2^nd^ trace of day 5). Therefore, in spite of the reduced mitochondrial respiratory chain function of the *nuo-6* strain [13,24], the main patterns of [Ca^2+^]_M_ dynamics were similar to those in the controls. However, a more detailed analysis including data from 23-36 different worms of every age uncovered significant differences.

**Figure 5:**
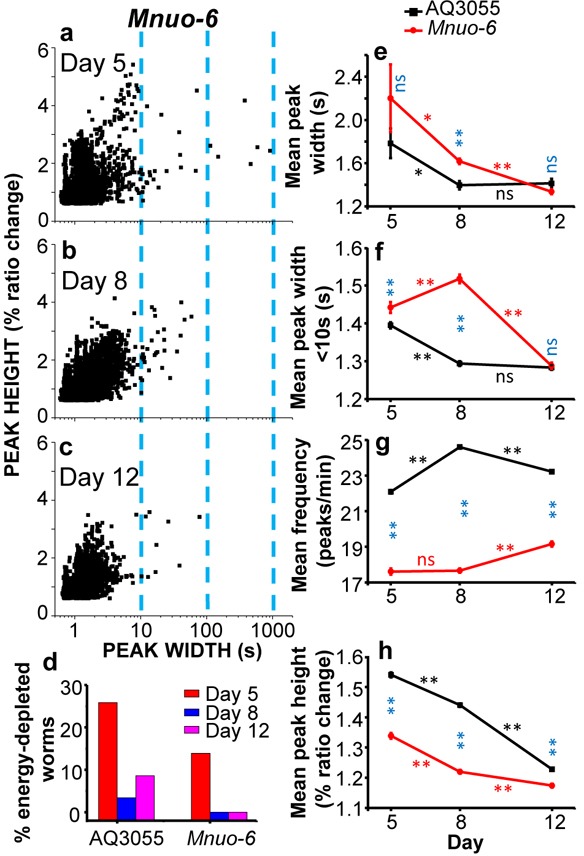
Comparative analysis of [Ca^2+^]_M_ dynamics in AQ3055 or *Mnuo-6* worms of 5, 8 and 12 days old Panels **a.**-**c.** show plots of peak height against peak width for all the [Ca^2+^]_M_ peaks analyzed in *Mnuo-6* worms of 5 (panel a, 4016 peaks from 36 worms studied), 8 (panel b, 5512 peaks from 28 worms studied) and 12 (panel d, 4430 peaks from 23 worms studied) days old. Panel **d.** shows the percentage of worms considered as energy-depleted in each case. The other panels **e.**-**h.** compare the evolution with age of the main parameters analyzed in both types of worms, AQ3055 and *Mnuo-6*: mean peak width (panel e), mean peak width considering only peaks with width < 10s (panel f), mean peak frequency (panel g) and mean peak height (panel h). Significance was obtained by the ANOVA test (*, *p* < 0,05; **, *p* < 0,001; ns, not significant) comparing: i) the control and mutant nuo-6 [Ca^2+^]_M_ data of the same day (blue symbols), ii) the control [Ca^2+^]_M_ data of every two consecutive days (black symbols) and iii) the mutant nuo-6 [Ca^2+^]_M_ data of every two consecutive days (red symbols).

Figure 5 shows a comparative analysis of the [Ca^2+^]_M_ peaks obtained in the AQ3055 (mitochondrial probe in control worms) and *Mnuo-6* strains (mitochondrial probe in *nuo-6* mutants). Panels a-c show the plots of peak height against peak width in *Mnuo-6* mutant worms of days 5, 8, and 12 of adult life. As in the controls (see Figure 3), the proportion of long “square-wave” [Ca^2+^]_M_ transients decreased with age, but the percent of worms categorized as energy-depleted was smaller at every age (Figure 5d). This is consistent with our previous [Ca^2+^]_C_ data in *nuo-6* worms [23], which also showed a reduced percentage of energy-depleted *nuo-6* worms compared with the controls at every age. On the other hand, the mean peak width of the [Ca^2+^]_M_ peaks was larger in the *nuo-6* mutant worms than in the controls at days 5 and 8, and similar at day 12 (Figure 5e). The same happened with the mean peak width calculated excluding the [Ca^2+^]_M_ peaks longer than 10s (Figure 5f), and these data follow exactly the same pattern we have previously described for the [Ca^2+^]_C_ peaks in the *nuo-6* mutant strain compared with the control [23]. Therefore, *nuo-6* worms have wider [Ca^2+^]_C_ and [Ca^2+^]_M_ peaks than controls at days 5 and 8 of adult life, and return to the control values at day 12. There were also significant differences in the frequency of the [Ca^2+^]_M_ peaks (Figure 5g), which was much smaller in *nuo-6* mutant worms than in the controls at every age, as occurs also with the frequency of the [Ca^2+^]_C_ peaks [23]. Finally, the height of the [Ca^2+^]_M_ peaks, was also smaller in *nuo-6* mutant worms than in the controls at every age (Figure 5h).

### Age-dependence of the frequency of pharynx [Ca^2+^]M oscillations

**Figure 6:**
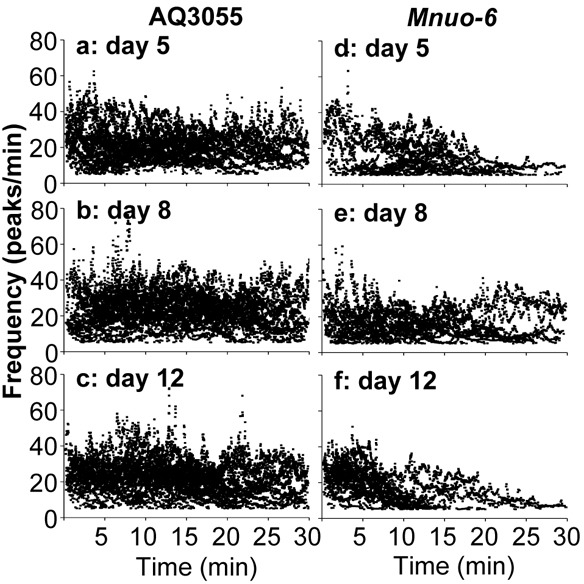
Evolution of the [Ca^2+^]_M_ oscillation frequency along the experiments Each point indicates the individual frequency value assigned to each peak as described in Methods, and the time point corresponding to that peak within the experiment. Panels **a.**-**c.** correspond to AQ3055 worms of 5, 8 and 12 days old, respectively. Panels **d.**-**f.** correspond to *Mnuo-6* worms of 5, 8 and 12 days old, respectively.

The dynamics of [Ca^2+^]_M_ spiking along the experiments is better appreciated by looking at the variation of the frequency of oscillation during the 30 minutes of experiment. Figure 6 shows the individual frequency data accumulated for all the worms studied of every age. Each point represents the frequency value assigned to each peak, as described in Methods, and the time the peak appeared within the 30 min experiment. Panels a-c correspond to the [Ca^2+^]_M_ data obtained in control AQ3055 worms, and show that the frequency kept quite stable for the whole time of the experiment, with maximum frequencies near 60 peaks/min. Therefore, mitochondria were able to maintain high frequencies of [Ca^2+^] oscillation for very long times *in vivo*. In the case of the *Mnuo-6* mutant worms (panels d-f), it is apparent that the frequency was smaller than in the controls. Many worms were still able to keep oscillations until the end of the 30 minutes, as shown in Figure 4, but there was some decay in the frequency along the experiment. This was mainly due to some worms that stopped [Ca^2+^]_M_ oscillations before the end of the experiment. The same behavior was found for the [Ca^2+^]_C_ oscillations in *nuo-6* mutant worms (data not shown), indicating that the decay of the [Ca^2+^]_M_ oscillations may be just a consequence of the decay in the [Ca^2+^]_C_ oscillations. In these cases, when [Ca^2+^] oscillations stop, [Ca^2+^]_C_ and [Ca^2+^]_M_ stay at resting levels and thus it cannot be attributed to energy depletion in the pharynx, but to some other reason that affects transmission of the pumpìng signal to the pharynx.

## DISCUSSION

As far as we know, the data we present in this paper constitute the first measurements of the dynamics of mitochondrial [Ca^2+^] in the pharynx of live *C. elegans* worms. Our data show the behavior of *C. elegans* pharynx [Ca^2+^]_M_ dynamics for long periods of time, and how it may depend on the worm age and on the presence of a respiratory chain complex I mutation. Mitochondrial [Ca^2+^] has been measured previously in worm hypodermis [25].

The first conclusion we can draw from our data is that mitochondrial [Ca^2+^] is able to oscillate at high frequency, reproducing the oscillations of cytosolic [Ca^2+^]. We have recorded frequencies of [Ca^2+^]_M_ oscillation up to 1Hz. This point is important because the *C. elegans* pharynx is a neuromuscular organ which has been compared to the heart because it has similar electrical properties and its development is controlled by similar genes [26]. Mitochondrial [Ca^2+^] signaling in the heart is controversial because there are now two different models to explain how the [Ca^2+^]_C_ oscillations are translated to mitochondria. One of the models suggests that mitochondria rapidly follow “beat-to-beat” the oscillations in [Ca^2+^]_C_, while the other model proposes that [Ca^2+^]_M_ only reflects a slow integration of the [Ca^2+^]_C_ transients [27]. The two models predict very different [Ca^2+^]_M_ kinetics in response to the repetitive cytosolic [Ca^2+^] spikes, either repetitive [Ca^2+^]_M_ spikes in the first model or a slow progressive increase in the second. Our data clearly show that the *C. elegans* pharynx mitochondria behave in a “beat-to-beat” mode, indicating that Ca^2+^ uptake and release from the mitochondria are fast enough to recover the resting levels after each Ca^2+^ spike. This point has several physiological implications. On the first place, energy consumption is higher with this model, because both Ca^2+^ uptake and release by mitochondria consume mitochondrial membrane potential. In addition, larger amounts of Ca^2+^ are taken up by mitochondria during each [Ca^2+^]_c_ spike, so that mitochondrial Ca^2+^ buffering may potentially become an important regulator of cytosolic [Ca^2+^] and thus, of contraction.

When we examine in detail the parameters of the [Ca^2+^]_M_ peaks, we find that they show a smaller width and frequency with respect to the [Ca^2+^]_C_ peaks. Considering only the [Ca^2+^] peaks having a width smaller than 10s at all ages, the peak width of the [Ca^2+^]_M_ peaks was 11.1±0.6% (mean±s.e.) smaller than that of the corresponding [Ca^2+^]_C_ peaks. Similarly, pooling all the frequency data at every age together, the frequency of the [Ca^2+^]_M_ peaks was 17.3±0.5% (mean±s.e.) smaller than that of the corresponding [Ca^2+^]_C_ peaks. In both cases, the difference was highly significant (*p* < 0.001). This may be due to the low Ca^2+^-affinity of the mitochondrial Ca^2+^ uniporter [20, 28], that may open only when [Ca^2+^]_c_ is near the peak levels, thus reducing the time mitochondrial Ca^2+^ uptake is active. The decrease in frequency could also be attributed to the presence of [Ca^2+^]_C_ peaks too small to evoke any mitochondrial Ca^2+^ uptake. On the other hand, we find that the [Ca^2+^]_M_ oscillation frequency hardly changes during aging, but there is however a progressive reduction in the width and amplitude of the [Ca^2+^]_M_ peaks with the age. Our data therefore indicate that mitochondrial Ca^2+^ fluxes in *C. elegans* pharynx are progressively reduced during aging, although mitochondria keep able to follow “beat-to-beat” the cytosolic [Ca^2+^] oscillations.

Together with the fast [Ca^2+^]_M_ oscillations, we could also find prolonged elevations of [Ca^2+^]_M_ similar to the “square-wave” [Ca^2+^]_C_ increases we have recently reported [23]. Many of them also lasted for many minutes and sometimes [Ca^2+^]_M_ stayed high for the rest of the experiment. The proportion of worms undergoing “square-wave” [Ca^2+^]_C_ or [Ca^2+^]_M_ increases decayed in parallel with the age, suggesting that they are both directly linked. Our hypothesis is that these long [Ca^2+^] transients can be attributed to energy depletion, which would arrest Ca^2+^ pumps and thus keep [Ca^2+^]_C_ high, leading then also to mitochondrial Ca^2+^ accumulation. If energy depletion is transient, cells may recover and restart spiking, but if it is persistent, the increased [Ca^2+^]_M_ may induce cellular damage by triggering ROS production or by activating apoptotic pathways. Energy depletion would be a consequence of the increased energy consumption during the experiment due to serotonin-induced accelerated pharynx pumping. The meaning of these “square-wave” [Ca^2+^]_C_ or [Ca^2+^]_M_ increases would be therefore that in these worms energy production is unable to cope with the increased energy consumption at some point during the 30 min of the experiment.

*Nuo-6* worms have a mutation in the respiratory chain complex I that produces reduced mitochondrial function, lower oxygen consumption, slow growth, slow movement [24] and decreased ATP levels [13]. In spite of these functional defects, mitochondria were able to display beat-to-beat [Ca^2+^]_M_ oscillations coupled to the [Ca^2+^]_C_ dynamics also in these worms. However, [Ca^2+^]_M_ oscillations in *nuo-6* mutant worms were slightly wider, and had smaller amplitude and frequency than in the controls, suggesting reduced Ca^2+^ fluxes in and out of the mitochondria. *Nuo-6* worms also had a reduced number of “square-wave” [Ca^2+^]_C_ and [Ca^2+^]_M_ increases at every age. This phenomenon could be due to the smaller rate of energy consumption in these mutants, which may balance the decreased rate of energy production.

## MATERIALS AND METHODS

### *C. elegans* strains and maintenance

Strains used were: AQ2038, integrated strain expressing cytosolic yellow cameleon 2.1. (YC2.1) on pharynx cells due to the promoter sequence of the *myo-2* gene (pmyo-2::YC2.1) [22, 29, 30]. AQ3059, strain expressing cytosolic yellow cameleon 3.60 (YC3.60) as extrachromosomal array on pharynx, under the myo-2 promoter (pmyo-2::YC3.60). AQ3055, strain expressing mitochondrially-targeted yellow cameleon 3.60 (YC3.60) as extrachromosomal array on pharynx, also under the myo-2 promoter (pmyo-2::2mt8::YC3.60). A signal peptide composed of the first 36 amino acids of subunit VIII of human COX, inserted in duplicate before YC3.60 [31], was used for mitochondrial targeting. Mutant *nuo-6* strains expressing the mitochondrially-targeted YC3.60 (named *Mnuo-6* strain) were obtained by crossing AQ3055 worms with *nuo-6* (qm200) ones [24], obtained from the Caenorhabditis Genetics Center.

Handling of the worms was as previously described [32]. All the strains were grown at 20°C on hardened agar seeded with Escherichia coli (OP50). Calcium imaging experiments were also carried out at 20°C on synchronized worms. Eggs obtained as described previously [32] were transferred to OP50-seeded NGM plates. For synchronization, young adults (day 1) were transferred to plates containing 15μM FUDR to avoid progeny.

### Calcium imaging

Pharynx Ca^2+^ measurements were carried out as previously described [23], at days 2, 5, 8 and 12 of worm life. Briefly, worms were starved for 4 - 6 hours before the experiments. Then, they were glued (Dermabond Topical Skin Adhesive, Johnson & Johnson) on an agar pad (2% agar in M9 buffer) made on a coverslip, and 5mg/ml of serotonin was added to stimulate pumping [23, 33, 34]. The coverslip containing the glued worm was mounted in a chamber in the stage of a Zeiss Axiovert 200 inverted microscope. Fluorescence was excited at 430 nm using a Cairn monochromator (4nm bandwidth for the cytosolic Ca^2+^ sensor in AQ2038, 7 nm bandwidth for the mitochondrial sensor in AQ3055 and *Mnuo-6*, continuous excitation) and images of the emitted fluorescence obtained with a Zeiss C-apochromat 40×1,2 W objective were collected using a 450nm long pass dichroic mirror and a Cairn Optosplit II emission image splitter to obtain separate images at 480nm and 535nm emission. The splitter contained emission filters DC/ET480/40m and DC/ET535/30m, and the dichroic mirror FF509-FDi01-25×36 (all from Chroma Technology). Simultaneous 200ms images at the two emission wavelengths were recorded continuously (2.5Hz image rate) by a Hamamatsu ORCA-ER camera, in order to obtain 535nm/480nm fluorescence ratio images values of a region of interest enclosing the pharynx terminal bulb. The time resolution used here is therefore higher (2.5Hz) than that used previously (1.5Hz, see [23] Alvarez-Illera et al., 2016), and that allowed recording higher frequencies of oscillation. Thus, the cytosolic [Ca^2+^] measurements included here correspond to new experiments performed in the AQ2038 strain at 2,5Hz time resolution. Experiments were performed at 20°C and carried on during 30 minutes of continuous recording.

For confocal imaging, worms anesthetized with 10mM tetramisole were imaged on a Leica TCS SP5 confocal microscope. YC3.60 fluorescence was excited at 488nm, and the fluorescence emitted between 500 and 554 nm was collected. Mitotracker Deep Red FM (Invitrogen, Catalog number M22426) was excited at 644nm, and the fluorescence emitted between 657 and 765nm was collected. Mitotracker deep red was loaded by incubating the worms for 2h with 10μM of the dye in M9 buffer.

### Data analysis

Fluorescence was recorded and analyzed using the Metafluor program (Universal Imaging). The traces shown are the 535nm/480nm fluorescence emission ratio values of a region of interest enclosing the pharynx terminal bulb. [Ca^2+^] peaks in the ratio were only considered acceptable when inverted changes at both wavelengths were clearly observed. Fluorescence intensities and ratio changes were then analyzed with a specific algorithm designed to calculate off-line the width at mid-height expressed in seconds, the height obtained as percent of ratio change and the frequency of all the Ca^2+^ peaks in each experiment. The frequency was measured at each peak as 9 divided by the distance among the peak 4 positions before and the peak 4 positions after. The mean frequency was calculated as the mean of all the individual frequencies higher than 5 peaks/min obtained in worms of a given age and condition.
